# Glucose fluctuation promotes cardiomyocyte apoptosis by triggering endoplasmic reticulum (ER) stress signaling pathway *in vivo* and *in vitro*

**DOI:** 10.1080/21655979.2022.2080413

**Published:** 2022-06-15

**Authors:** Li-Da Wu, Ying Liu, Feng Li, Jia-Yi Chen, Jie Zhang, Ling-Ling Qian, Ru-Xing Wang

**Affiliations:** Department of Cardiology, Wuxi People’s Hospital Affiliated to Nanjing Medical University, Wuxi, Jiangsu, China

**Keywords:** Glucose fluctuation, cardiomyocyte apoptosis, ER stress, 4-PBA

## Abstract

Glucose fluctuation is more harmful than sustained hyperglycemia, but the effect on cardiomyocyte apoptosis have not yet been clarified. In this study, we aim to identify the effect of glucose fluctuation on cardiomyocyte apoptosis and explore the underlying mechanism. Sprague-Dawley rats were intraperitoneally injected with streptozotocin (STZ) and divided into three groups: controlled diabetic group (C-STZ); uncontrolled diabetic group (U-STZ) and glucose fluctuated diabetic group (GF-STZ). After twelve weeks, echocardiography, Hematoxylin-eosin (HE) staining, and Masson staining were adopted to assess the cardiac function and pathological changes. TUNEL staining was used to detect apoptotic cells. Expressions of apoptosis-related proteins and key molecules in the endoplasmic reticulum (ER) stress pathway were determined via western blots. Further, primary cardiomyocytes incubated in different glucose conditions were treated with the inhibitor of ER stress to explore the causative role of ER stress in glucose fluctuation-induced cardiomyocyte apoptosis. *In vivo*, we demonstrated that glucose fluctuation promoted cardiomyocyte apoptosis, and were more harmful to cardiomyocytes than sustained hyperglycemia. Moreover, glucose fluctuation significantly triggered ER stress signaling pathway. *In vitro*, primary cardiomyocyte apoptosis induced by glucose fluctuation and the activation of ER stress were significantly attenuated by 4-PBA, which is an ER stress inhibitor. Above all, glucose fluctuation can promote cardiomyocyte apoptosis through triggering the ER stress signaling pathway in diabetic rats and in primary cardiomyocytes.

## GRAPHICAL ABSTRACT

**Figure:**
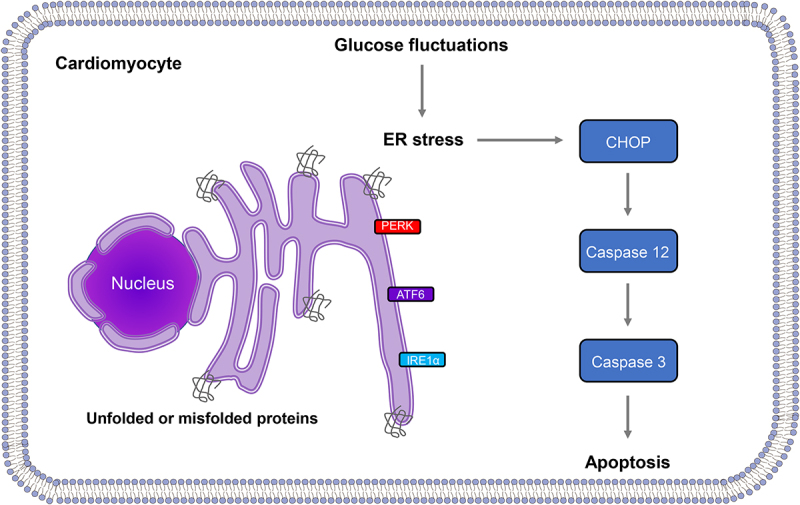

## Highlights

- Glucose fluctuation promotes cardiac dysfunction in diabetes;
- Glucose fluctuation leads to ER stress-induced cardiomyocyte apoptosis;
- 4-PBA can effectively reduce cardiomyocyte apoptosis induced by glucose fluctuation;
- This study highlights a potential therapeutic target for diabetic cardiomyopathy.

## Introduction

Diabetes has become a major public health concern worldwide, which is responsible for 6.7 million deaths in 2021. The number of patients with diabetes has increased to 537 million, even worse, diabetes is spiraling out of control. International Diabetes Federation estimated that there will be more than 780 million patients with diabetes in 2045 [1]. Diabetes can cause damage to heart, brain, kidney, peripheral nerve, retina, and other important target organs. Diabetic cardiovascular complications are important reasons for the increase of mortality in patients with diabetes. Diabetes is associated with increased risk of cardiac dysfunction regardless of the existing of hypertension, coronary artery disease, and valvular heart disease, named diabetic cardiomyopathy. On the basis of metabolic disorder, diabetes cardiomyopathy leads to extensive focal necrosis of myocardium and eventually progresses to heart failure, cardiogenic shock, and even sudden cardiac death in severe patients. It is necessary to further study the underlying molecular mechanisms of diabetic cardiomyopathy.

Hyperglycemia in patients with diabetes can be manifested as chronic persistent hyperglycemia and chronic fluctuating hyperglycemia (glucose fluctuation). Glucose fluctuation refers to the unstable state of blood glucose level changing between peak and trough due to poor blood glucose control. Recently, many investigations have shown that glucose fluctuation has a more serious impact on cardiovascular disease than sustained hyperglycemia, leading to hypertension and coronary artery systolic and diastolic dysfunction in coronary artery disease [2–4]. Moreover, Saito *et al*. reported that glucose fluctuation can increase the incidence of atrial fibrillation by promoting cardiac fibrosis [5]. Therefore, it is of great significance to strengthen the blood glucose management of patients with diabetes and avoid blood glucose fluctuation (especially postprandial blood glucose fluctuation) to reduce the complications of diabetes. However, whether glucose fluctuation exacerbates diabetic cardiomyopathy and the damage mechanisms of the cardiac function due to glucose fluctuation remain elusive.

Endoplasmic reticulum (ER) is an organelle that exists in most eukaryotes. It is mainly involved in the synthesis of secretory proteins and the regulation of intracellular calcium balance [6]. Adverse environmental challenges, such as nutrient depletion, oxidative stress, and pH changes, can lead to misfolded and unfolded proteins accumulated in the ER, and eventually ER stress [7]. In order to reduce unfolded and misfolded proteins, ER stress sensors are activated to accelerate protein degradation and reduce the synthesis of new proteins, called unfolded protein reaction. Under ER stress, cells firstly elicit adaptive responses by enhancing protein-folding capacity and accelerating the degradation of unfolded proteins. Immunoglobulin-binding protein, also known as glucose-regulated protein 78 (GRP78), is up-regulated to increase the protein-folding capacity during ER stress. However, chronic and severe ER stress can ultimately lead to apoptosis [8]. Apoptosis plays an important role in diabetic cardiovascular complications [9]. Since cardiomyocytes cannot proliferate, the loss of cardiomyocytes directly contributes to contractile unit loss, consequently leading to the development of systolic and diastolic dysfunction [10]. Accumulated studies have indicated that pathological increase of cardiomyocytes apoptosis can worsen the prognosis of patients with heart failure [11–13]. C/EBP homologous protein (CHOP) normally expresses very low. During ER stress, CHOP is up-regulated and increases the expression of Caspase 12, which is secreted into the cytoplasm from ER to further activate Caspase 3 and eventually leads to apoptosis [14]. ER stress-induced apoptosis is involved in cancers, neurodegenerative disorders, retinal degeneration, respiratory infections, metabolic diseases, etc. [15]. Younce et al. indicated that reducing ER stress-induced apoptosis could provide cardioprotective effects [16]. Nevertheless, it has not been reported whether glucose fluctuation can enhance ER stress and its mediated apoptosis, finally aggravate diabetes cardiomyopathy.

The aim of this study was to reveal the underlying molecular mechanisms by which glucose fluctuation contributes to myocardial injury. We hypothesized that glucose fluctuation can enhance ER stress and eventually lead to cardiomyocyte apoptosis. A diabetic rat model *in vivo* and cardiomyocytes treated with an ER-stress inhibitor (4-Phenylbutyric acid, 4-PBA) *in vitro* were established to evaluate the potential effect of glucose fluctuation on myocardial apoptosis. We found that ER stress-induced cardiomyocyte apoptosis plays a crucial role in chronic cardiac dysfunction caused by blood glucose fluctuation. Here, we attempt to provide theoretical and experimental evidence for potential therapeutic targets for diabetic cardiomyopathy.

## Methods

### Experimental animal models

Healthy male Sprague-Dawley rats (180–200 g) were purchased from Jiangsu Institute of Schistosomiasis Control and Prevention, and then placed in pathogen-free condition with available water and food. Rats with type 1 diabetes were induced according to previously documented methods [4]. Briefly, intraperitoneal injection of STZ in rats leads to the destruction of islet cells, leading to type 1 diabetes. One week later, blood glucose of rats was measured in the tail vein. If glucose was >16.7 mmol/L, the rat was enrolled. Rats were assigned to three groups randomly: controlled diabetic rats (C-STZ), uncontrolled diabetic rats (U-STZ), and glucose fluctuated diabetic rats (GF-STZ). At 8 a.m. every morning and 8 p.m. every evening for 12 weeks, C-STZ rats received therapy of subcutaneous injection of long-acting insulin (20 IU/kg). U-STZ rats were fed normally with adequate food. GF-STZ rats were alternatingly starved for 24 hours followed by a 24 hours period with adequate food to induce blood glucose fluctuation, as in previous studies.^4^ The islet functions of STZ-induced diabetic rats are extremely poor. Therefore, after 24 hours of exposure to adequate food, glucose levels of the GF-STZ rats were always >25.5 mmol/L. However, if the glucose levels exceeded 5.5 mmol/L after 24 hours of starvation, the GF-STZ rats would be subcutaneously injected with short-acting subcutaneous insulin (0.5 IU/kg) to control it. After 12 weeks, rats were sacrificed and hearts were isolated from chests, and stored at −80°C for experiments. Body weight and blood glucose were measured in a fixed time every day (between 12:00 p.m. and 1:00 p.m.). All protocols involving the use of animal subjects were approved by the Institutional Animal Care and Use Committee of Nanjing Medical University (IACUC-1712028).

### Echocardiography examination

After 12 weeks of modeling, the rats were anesthetized with inhalation of isoflurane (2%). Echocardiography was used to evaluate left ventricular function [17].

### Isolation of neonatal rat cardiomyocytes

Day 1–3 neonatal rat hearts were removed from their chests after disinfecting rats’ bodies with 75% ethanol. Neonatal hearts were placed into 1.5 mL sterile tubes with 500 μL phosphate buffered saline (PBS, Gibco., #10010049). Hearts were minced into 1 mm^3^ pieces. We washed the minced tissue twice with 500 μL PBS to remove blood from the tissues. An appropriate amount of myocardial enzyme mixture (Pierce Primary Cardiomyocytes Isolation Kit, Thermo., #88281) was added into tubes. Then, we incubated tubes at 37°C. After 35 minutes, the tissues were washed twice with PBS. DMEM (1 g/L D-Glucose, Gibco., #11885084) was added to each tube and the tissue was broken up by pipetting up and down 25–30 times. After centrifugation for 5 minutes at 1000 rpm, the neonatal rat cardiomyocytes were isolated, and then seeded and cultured in 6-well plates (37°C and 5% CO2) [18].

### *Glucose fluctuation model* in vitro

Establishment of the glucose fluctuation model *in vitro* was as reported previously [4]. In brief, neonatal rat primary cardiomyocytes were divided into three groups: the normal glucose (NG) group, the high glucose (HG) group, and the glucose fluctuation (GF) group. NG group cells were cultured in a low-glucose condition of 5.5 mmol/L, HG group cells were cultured in high-glucose condition of 25 mmol/L, while GF group cells were cultured alternately in 5.5 mmol/L and 25 mmol/L glucose conditions for 72 h, changing every 12 h. To confirm the effect of ER stress on apoptosis, the cardiomyocytes were treated with 2 mmol/L 4-PBA (an inhibitor of ER stress).

### Hematoxylin-eosin (HE) staining

The fresh rat hearts were sliced into sections of appropriate thickness. The heart sections were fixed with 4% paraformaldehyde (Beyotime Biotechnology Co., #P0099-500), dehydrated with gradient ethanol, waxed, and embedded with paraffin. They were then cut into a thickness of 5 μm and stained with HE solution (Beyotime Biotechnology Co., #C0105). Sections were examined under optical microscope [19].

### Masson staining

A 5 μm thick section of heart tissue was stained with Masson-staining solution (Nanjing Jiancheng Bioengineering Institute, #D026). Slices were taken under a 40x objective lens [2].

### Immunofluorescence analysis

After deparaffinization and rehydration, paraffin sections were treated with anti-CHOP antibody (Cell Signaling Technology Co., #2895) and DAB solution (ZsBio Store Co., #ZLI-9018). All pieces were counterstained with hematoxylin (Beyotime Biotechnology Co., #C0107) and sealed with neutral balsam (HUSHI Co., #10004160). Slices were taken under a 20x objective lens [20].

### TUNEL staining

Masses of freshly isolated rat hearts were fixed with 4% paraformaldehyde for 24 h, and then immersed in 15% (gram/ml) and 30% (gram/ml) sucrose solution, in turn, until they settled to the bottom. Dehydrated heart tissues were embedded by OCT (Sakura Bio Co., #4583) and sliced into 4 μm thick sections. After fixed with 4% paraformaldehyde for 10 min and permeated with freshly prepared 0.1% Triton X-100 (Beyotime Biotechnology Co., #P0096), the sections were combined with freshly prepared TUNEL reaction solution (Roche Co., #11684795910) in a dark room and incubated for 1.5 h. Finally, DAPI (Sigma-Aldrich Co., #F6057) was added to the tissue to show the nucleus. Images were taken under a confocal microscope [21].

### Western blot

Heart tissue was taken from −80°C storage and cut to appropriate size, soaked in RIPA buffer (Thermo Fisher Scientific Co., #89901) containing phosphatase inhibitor and protease inhibition (Thermo Fisher Scientific Co., #78442), and fully cut into pieces. We used BCA Protein Assay Kit (Thermo Fisher Scientific Co., #23227) to determine the protein concentration and adjust it to a uniform level. Then, the expression of the target protein was measured by Western blotting. PVDF membranes were incubated with specific primary antibodies overnight, including CHOP (Cell Signaling Technology Co., Mouse mAb, #2895), GRP78 (Cell Signaling Technology Co., Rabbit pAb, #3183), Caspase 12 (Abcam Bio. Co., Rabbit pAb, #ab62484), Cleaved Caspase 3 (Cell Signaling Technology Co., Rabbit pAb, #9661), Bax (Cell Signaling Technology Co., Rabbit pAb, #2772), Bcl-2 (Abcam Bio. Co., Rabbit pAb, #ab196495) and GAPDH (Cell Signaling Technology Co., Rabbit mAb, #5174) following manufacturer’s suggested protocols. Then, the corresponding secondary antibodies were incubated according to the source of primary antibodies, which were mouse and rabbit antibodies, respectively. Finally, the results were quantified by ImageJ software (Scion Corp., USA) [22].

### Statistical analysis

Data are presented as the mean ± SEM. Statistical analysis was performed with SPSS 25.0. Each group of data was subjected to normality and homogeneity of variance testing. For comparison of three or more groups, one-way ANOVA was used, and pairwise comparisons between groups were performed with least significant difference (LSD) or Student-Newman-Keuls (SNK) tests. A *P* value of less than 0.05 was considered statistically significant [23].

## Results

Glucose fluctuation is an unstable state, in which blood glucose changes between peaks and valleys due to poor blood glucose control. In the present study, we hypothesized glucose fluctuation could enhance the accumulation of unfolded proteins and misfolded proteins in the ER and promote ER stress-induced apoptosis, eventually aggravate cardiac dysfunction in diabetes. To prove this hypothesis, we established diabetic rat models and cultured primary cardiomyocytes in different glucose concentrations. Echocardiography, pathological staining, and molecular biological methods were adopted to investigate effect of glucose fluctuation on myocardial injury and its underlying mechanism, providing new ideas for the treatment of diabetic cardiomyopathy.

### Glucose fluctuation exacerbates cardiac dysfunction

**Figure 1.**
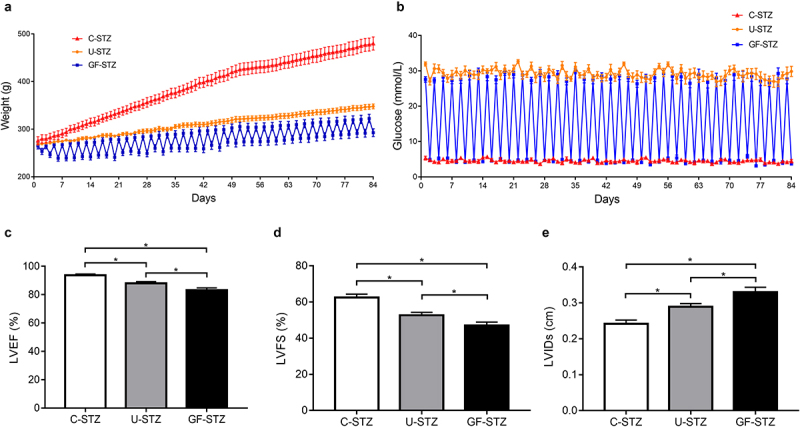
The blood glucose level and body weight in diabetic rats (a) The blood glucose level over 12 weeks in C-STZ, U-STZ and GF-STZ groups. (b) The body weight over 12 weeks in different groups. (c-e) The analysis of left ventricular EF, FS and LVIDs in different groups. C-STZ: controlled diabetic group; U-STZ: uncontrolled diabetic group; GF-STZ: glucose fluctuated diabetic group. LVFS: left ventricular fractional shortening; LVEF: left ventricular ejection fraction. LVIDs: left ventricular end systolic diameter. n = 8 per group, *P < 0.05.

Blood glucose of rats in the C-STZ and U-STZ groups were maintained at about 5.5 mmol/L and 25 mmol/L respectively, while the blood glucose of rats in the GF-STZ group fluctuated in a wide range between 5.5 and 25 mmol/L (Figure 1(a)). At the initial stage of modeling, the weight of rats in the three groups was similar. However, with the extension of time, the weight of rats in the C-STZ group became significantly higher than that in the U-STZ and GF-STZ groups (Figure 1(b)). Cardiac function of rats was evaluated by cardiac ultrasonography. As can be seen from Figure 1(c-e) and Table 1, LVEF and LVFS were significantly decreased and LVIDs was significantly increased in the GF-STZ group, and there was also a statistically significant difference between the U-STZ and GF-STZ rats, indicating that glucose fluctuation can significantly aggravate the cardiac systolic dysfunction in diabetic rats.

**Table 1.** Echocardiographic analysis in different groups

	C-STZ	U-STZ	GF-STZ	F value	*p* value
LVEF %	93.69 ± 2.45	87.93 ± 3.57*	83.11 ± 4.68^*#^	16.56	0.00
LVFS %	62.54 ± 5.18	52.73 ± 4.55*	47.00 ± 5.38^*#^	19.38	0.00
LVPWs (cm)	0.26 ± 0.03	0.24 ± 0.03	0.24 ± 0.02	2.58	0.10
LVIDs (cm)	0.24 ± 0.03	0.29 ± 0.02*	0.33 ± 0.04^*#^	15.55	0.00
IVSs (cm)	0.25 ± 0.04	0.22 ± 0.02	0.22 ± 0.27	1.92	0.17
LVPWd (cm)	0.15 ± 0.01	0.14 ± 0.02	0.15 ± 0.02	0.75	0.49
LVIDd (cm)	0.66 ± 0.10	0.62 ± 0.06	0.62 ± 0.06	0.64	0.54
IVSd (cm)	0.14 ± 0.02	0.14 ± 0.01	0.15 ± 0.02	0.09	0.91

n = 8 for each group. LVEF, ejection fraction; LVFS, fractional shortening; LVPWs, left ventricular posterior wall thickness at end systole; LVIDs, left ventricular internal dimension at end systole; IVSs, interventricular septal thickness at end systole; LVPWd, left ventricular posterior wall thickness at end diastole. LVIDd, left ventricular internal dimension at end diastole; IVSd, interventricular septal thickness at end diastole. *P < 0.05 vs. C-STZ. ^#^P < 0.05 vs. U-STZ.

### *Glucose fluctuation aggravates cardiac structural disorder and myocardial fibrosis* in vivo

**Figure 2.**
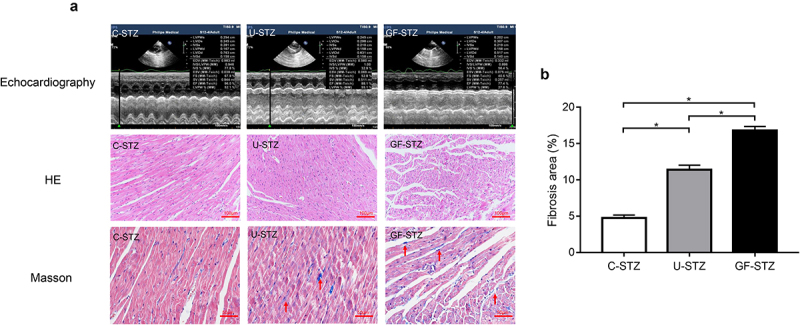
Effect of glucose fluctuation on cardiac structural disorder and myocardial fibrosis in *vivo* Representative images of echocardiography representative images of tissue sections of left ventricular stained with HE and Masson staining. C-STZ: controlled diabetic group; U-STZ: uncontrolled diabetic group; GF-STZ: glucose fluctuated diabetic group.

The above results have shown that glucose fluctuation can significantly reduce the systolic function of diabetic rats. To further explore the influence of blood glucose fluctuation on myocardial structure, HE and Masson staining were performed. The results of HE staining showed that the morphological structure of myocardial tissue in the U-STZ rats was significantly disorganized compared with C-STZ rats, and that the GF-STZ rats were even more disorganized. We also observed more deposition of collagen fibers in the myocardial tissues of GF-STZ rats by Masson staining (Figure 2). Therefore, glucose fluctuation intensifies heart structural disorder and significantly aggravate the occurrence of myocardial fibrosis in diabetic rats.

### Glucose fluctuation promotes the occurrence of cardiomyocyte apoptosis

**Figure 3.**
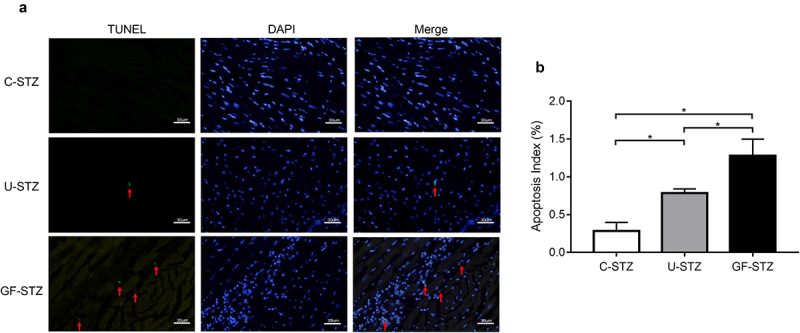
Glucose fluctuation promotes cardiomyocyte apoptosis (a) Representative images of tissue sections of left ventricular stained with TUNEL. (b) The quantitative ratio of apoptotic cells. TUNEL: apoptotic myocardial nucleus stained with TUNEL staining solution; DAPI: myocardial nucleus stained with DAPI staining solution; Merge: merged images of TUNEL staining and DAPI staining; C-STZ: controlled diabetic group; U-STZ: uncontrolled diabetic group; GF-STZ: glucose fluctuated diabetic group. n = 5 per group, *P < 0.05.

**Figure 4.**
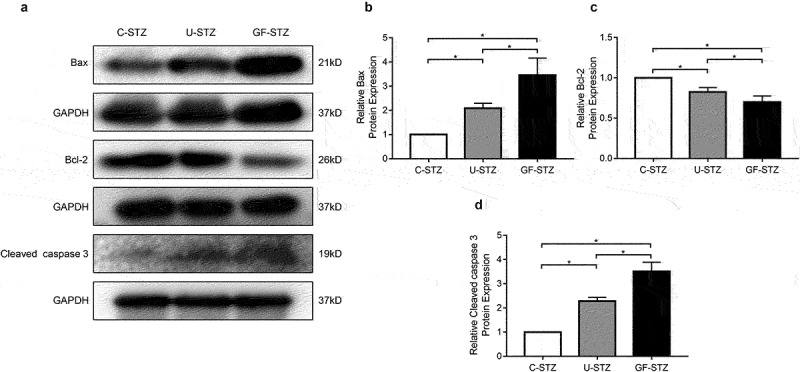
The protein expression of Bax, Bcl-2 and Cleaved Caspase 3 in rat hearts of different groups (a) Representative Western blotting bands of Bax, Bcl-2 and Cleaved Caspase 3. (b-d) Analysis of the expression of Bax, Bcl-2 and Cleaved Caspase 3 among different groups. C-STZ: controlled diabetic group; U-STZ: uncontrolled diabetic group; GF-STZ: glucose fluctuated diabetic group. n = 5 per group, *P < 0.05.

It is well known that diabetes can significantly promote the occurrence of cardiomyocyte apoptosis. According to the above results, GF-STZ group exhibits significantly aggravated disorder of cardiac structure, so we further explored the effect of glucose fluctuation on cardiomyocyte apoptosis. According to the results of TUNEL staining (Figure 3(a)), glucose fluctuation significantly increased genomic DNA fracture in the nucleus (as shown in the green fluorescence), which suggests the occurrence of cell apoptosis. We also detected the expression of apoptosis-related proteins. As shown in Figure 4. The results of the expression of Bax and Cleaved Caspase 3 were consistent with TUNEL staining. Protein expression of Bcl-2 in the U-STZ group was decreased compared to that in the C-STZ group, and the difference was more pronounced in the GF-STZ group. The above results suggest that glucose fluctuation significantly increases apoptosis of cardiomyocytes in diabetic rats, and to a greater degree than sustained hyperglycemia. Therefore, the occurrence of cardiac dysfunction under the condition of glucose fluctuation may be partly caused by the increase of pathological apoptosis of cardiomyocytes.

### Glucose fluctuation promotes ER stress signaling pathway

**Figure 5.**
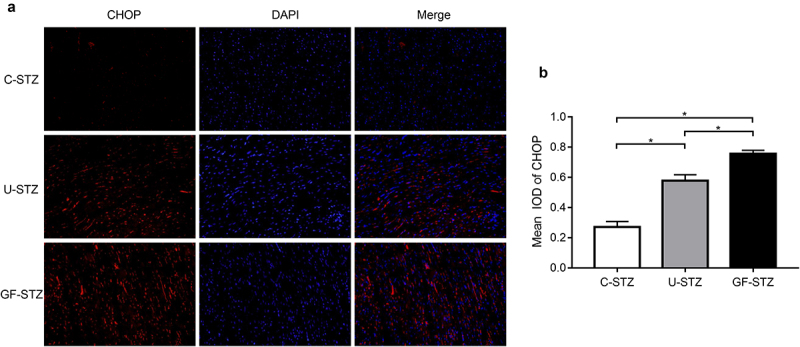
Effect of glucose fluctuation on CHOP expression in rat hearts of different groups (a) Representative CHOP immunofluorescence images of myocardial tissue sections of the left ventricle. (b) Integrated optical density of CHOP immunofluorescence images of different groups. C-STZ: controlled diabetic group; U-STZ: uncontrolled diabetic group; GF-STZ: glucose fluctuated diabetic group. n = 5 per group, *P < 0.05.

**Figure 6.**
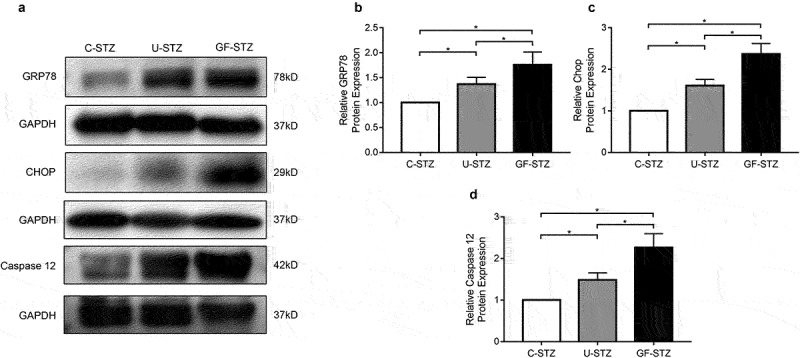
The protein expression of key markers of ER stress in rat hearts of different groups. (a) Representative Western blotting bands of GRP78, CHOP and Caspase 12 of different groups. (b-d) Analysis of the expression of GRP78, CHOP and Caspase 12 among different groups. C-STZ: controlled diabetic group; U-STZ: uncontrolled diabetic group; GF-STZ: glucose fluctuated diabetic group. n = 5 per group, *P < 0.05.

In order to explore the role of ER stress, and a downstream protein of ER stress, CHOP, in cardiomyocyte apoptosis caused by glucose fluctuation, we used heart tissues to detect the differences in its expression among the three groups by immunofluorescence. The expression of CHOP in the GF-STZ and U-STZ group was significantly increased, and to a greater degree in the GF-STZ group (Figure 5). Meanwhile, the relative expressions of ER stress-related proteins in the three groups were also evaluated at the protein level. The protein expression levels of CHOP, GRP78, and Caspase 12 in the GF-STZ group and the U-STZ group were significantly increased compared with that of the C-STZ group, and to greatest extent in the GF-STZ group (Figure 6). These results suggest that glucose fluctuation may promote ER stress and thus increase the apoptosis of cardiomyocytes.

### Inhibition of ER stress reduces cardiomyocyte apoptosis

**Figure 7.**
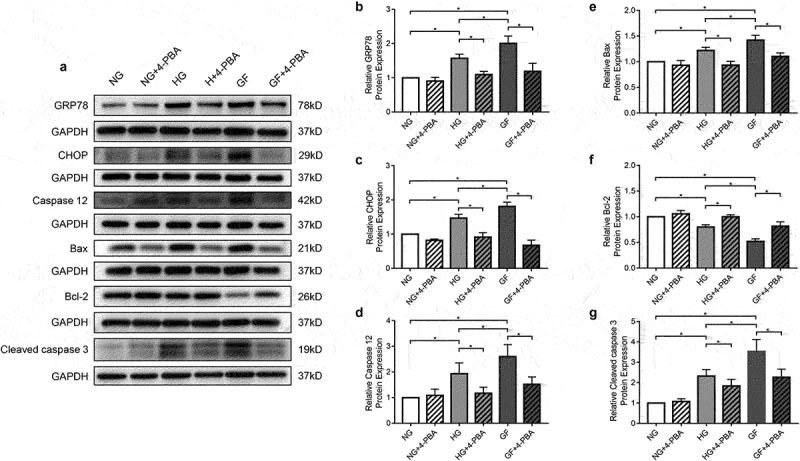
Role of ER stress specific inhibitor 4-PBA in glucose fluctuation mediated primary cardiomyocyte apoptosis in *vitro* (a) Western blots of primary cardiomyocytes incubated for 72 h in DMEM in absence or presence of 4-PBA (2 mmol/L). (b-d) Analysis of key marker molecules of ER stress (GRP78, CHOP and Caspase 12) in different groups, with and without 4-PBA. (e-g) Analysis of apoptotic protein markers (Bax, Bcl-2 and Cleaved Caspase 3), with and without 4-PBA.NG: normal glucose group; HG: high glucose group; GF: glucose fluctuated group. n = 5 per group, *P < 0.05.

As shown in Figure 7, 4-PBA, a specific inhibitor of ER stress, can significantly reduce the expression of CHOP, GRP78, and Caspase 12, in each treatment group. Moreover, after 4-PBA intervention on the primary cardiomyocytes, Bax, and Cleaved Caspase 3 expressions were significantly decreased in each group compared with their respective nonintervention group, while Bcl-2 expression was significantly increased. Thus, 4-PBA can significantly reduce apoptosis of cardiomyocyte under hyperglycemia and glucose fluctuation, which proves that glucose fluctuation may partly promote the activation of ER stress and thus cause increased cardiomyocyte apoptosis.

## Discussion

In this study, we firstly explored the effect of glucose fluctuation on diabetic cardiomyopathy and the underlying mechanism. The main findings are as follows: (1) glucose fluctuation promoted cardiac dysfunction and aggravated cardiomyocyte apoptosis more severely in diabetic rats compared with sustained hyperglycemia; (2) glucose fluctuation enhanced ER stress and increased the expression of key molecules of ER stress *in vivo* and *vitro*; (3) ER stress inhibitors (4-PBA) provided protective effects for glucose fluctuation-induced cardiomyocyte apoptosis.

Compared with normal individuals, patients with diabetes have increased cardiovascular morbidity and mortality. Diabetic cardiomyopathy, defined as cardiac dysfunction independent of coronary heart disease, hypertension, and other cardiovascular diseases, is the leading cause of death in patients with diabetes. Nevertheless, the exact molecular mechanisms of the development of diabetic cardiomyopathy still remain exclusive and are thought to be the result of oxidative stress and inflammation, as well as cardiomyocyte apoptosis caused by various metabolic causes. Liu *et al*. have reported that diabetes can lead to oxidative stress mediated myocardial fibrosis, and eventually cardiac dysfunction in the diabetic rat model, which plays an important role in the occurrence of diabetes cardiomyopathy [24]. Moreover, it has been reported in the literature that inhibiting inflammatory response and improving the immune microenvironment of myocardial tissue can alleviate cardiac dysfunction caused by diabetes [25]. At present, there are few treatment measures for patients with diabetic cardiomyopathy, and it is difficult to achieve satisfactory therapeutic results. Therefore, it is of clinical significance to further study the mechanisms of the diabetic cardiomyopathy and develop effective methods that can prevent and treat diabetic cardiomyopathy.

Glucose fluctuation often occurs in patients with poor blood glucose management. Based on 24 hour-continuous blood glucose monitoring machine, results of a clinical study showed that there is a significant glucose fluctuation in patients with poor blood glucose management, although the HbA1c values of some patients are excellent [26]. Recent studies, including our previous studies, have suggested that glucose fluctuation was more harmful than sustained hyperglycemia [2–5]. In addition to HbA1c, controlling of glucose fluctuation can provide protective effect for decreasing diabetic complications. Glucose fluctuation can lead to the destruction of homeostasis and activate multiple signaling pathways, eventually lead to the occurrence and development of diabetes complications. AKT is a crucial signal transduction intermediate, which is involved in the pathways of development of diabetic complications. Glucose fluctuation can lead to the reduction of phosphorylated AKT and eventually activate glycogen synthase kinase-3β (GSK-3β), which is an initiator of cell death [27]. In addition, Saito *et al*. demonstrated that glucose fluctuation exacerbated the level of oxidative stress compared with the sustained high glucose in the diabetic animal model, which contributed to the increase of TNF-α and TGF-β1 expression, leading to increased inflammation [5]. Nevertheless, the effect of glucose fluctuation on diabetic cardiomyopathy remains unknown. For the first time, this study investigated the underlying mechanisms by which glucose fluctuation contribute to myocardial injury.

ER is essential for homeostasis of the intracellular environment. Disturbances of ER homeostasis affect protein folding and contribute to an accumulation of misfolded proteins in the ER, ultimately lead to ER stress [28]. Many factors can contribute to ER stress, including metabolic diseases, inflammatory diseases, and neoplastic diseases [29]. Of note, accumulated evidence demonstrated that ER stress plays a pivotal role in the occurrence and development of various diabetic complications. It was found that sustained hyperglycemia can promote endoplasmic reticulum stress in a rat model of diabetic retinopathy, which contributes to activation of vascular endothelial growth factor signaling pathway and angiogenesis [30]. Su *et al*. also demonstrated that Icariin, a traditional Chinese medicine, can inhibit the ER stress response by promoting the expression of G protein-coupled estrogen receptors, reducing the proliferation of diabetic nephropathy [31]. However, the effect of glucose fluctuation on endoplasmic reticulum stress in diabetes has not been reported yet. We therefore hypothesized that glucose fluctuations might induce ER stress, leading to diabetic cardiomyopathy. During ER stress, GRP78 and CHOP is up-regulated to increase protein-folding capacity and activate molecular sensors of ER stress (IRE1α, ATF6, and PERK), leading to decreased accumulation of misfolded proteins and unfolded proteins [32]. GRP78 and CHOP have been selected as marker proteins of ER stress in a large number of studies on the mechanisms of ER stress in various diseases. Li *et al*. demonstrated that sulforaphane prevents rat cardiomyocytes from hypoxia/reoxygenation injury *in vitro* via inhibiting ER stress, in which GRP78 and CHOP was selected as marker proteins of ER stress [33]. Hosseini *et al*. investigated the role of ER Stress in a rat varicocele testis model, they also selected GRP78 and CHOP as marker proteins of ER stress [34]. In the present study, a diabetic rat model *in vivo* and cardiomyocytes treated with ER-stress inhibitors (4-PBA) *in vitro* were established to evaluate the potential effect of glucose fluctuation on ER stress. As anticipated, our data proved that GRP78 and CHOP greatly increased in both GF-STZ rats and primary cardiomyocytes with glucose fluctuation. Therefore, ER stress is indeed activated in the setting of glucose fluctuation.

Apoptosis is a process of autonomous extinction strictly regulated by cellular signals, which involves a series of gene activation, expression, and regulation [35]. The loss of cardiomyocytes resulting from excessive apoptosis when the cells are subjected to adverse conditions contributes to the development of systolic and diastolic dysfunction. Peng *et al*. revealed that the extent of cardiomyocyte apoptosis was related to the degree of cardiac dysfunction and clinical symptoms in diabetic cardiomyopathy [36]. Apoptosis occurs when the homeostasis of the ER is severely disturbed [37]. Of note, CHOP is the most important downstream component of ER stress-induced apoptosis. Caspase 12 is activated by CHOP and secreted into the cytoplasm to further activate Caspase 9, which cleaves pro-Caspase 3 and eventually leads to apoptosis. Activation of CHOP can promote apoptosis, and studies have shown that silencing CHOP can significantly reduce apoptosis induced by activation of ER stress [38]. Importantly, ER stress-induced apoptosis plays a crucial role in the progression of diabetic cardiovascular complications [39,40]. In our experiment, echocardiography was performed to evaluate cardiac function. Consistent with previous studies, cardiac function was worse in U-STZ group compared with C-STZ group, the GF-STZ group had the worst cardiac function. To further clarify whether the cardiac dysfunction induced by glucose fluctuation is related to cardiomyocyte apoptosis, we adopted TUNEL assay to investigate the extent of cardiomyocyte apoptosis. Our results suggested that the level of apoptosis was significantly increased in the U-STZ group, and the increase was most pronounced in the GF-STZ group. In addition, to fully dissect the role of glucose fluctuation on cardiomyocyte apoptosis, we tested the expression of Bcl-2, Bax, and Cleaved Caspase 3. Consistent with previous studies [41,42], we found that the levels of Bcl-2 were reduced and the levels of Bax and Cleaved Caspase 3 were increased in the U-STZ group, whereas glucose fluctuation exacerbated their trend. The above results indicated that glucose fluctuation has effect of promoting cardiomyocyte apoptosis. To further determine the role of ER stress in glucose fluctuation-induced pro-apoptotic activity, 4-PBA was used to treat primary cardiomyocytes. GRP78, CHOP, and Caspase 12 was reduced by the application of 4-PBA, and the degree of apoptosis was also decreased. Therefore, inhibition of endoplasmic reticulum stress signaling pathway can reduce cardiomyocyte apoptosis induced by blood glucose fluctuation, which may be a potential target for the treatment of diabetic cardiomyopathy.

Above all, our results suggest that glucose fluctuation can promote cardiomyocyte apoptosis by triggering ER stress signaling pathway. In clinical treatment, the therapeutic effect can be achieved by reducing glucose fluctuation. It is well known that ER stress happens during diabetes and also plays a pivotal role in the development of diabetic complications. This might be good news for patients with diabetes. The direct inhibition of ER stress or the indirect regulation of ER stress by controlling glucose fluctuation may be a novel therapeutic method for diabetic cardiomyopathy. However, there are several limitations of the present study should be noted. The cost of long-term application of 4-PBA in animal models is currently very high, especially the construction time of our glucose fluctuation animal model is relatively long. The effect of inhibition of ER stress on myocardial injury has not been confirmed *in vivo*. Moreover, the underlying molecular mechanisms of glucose fluctuation aggravating diabetic cardiomyopathy still need to be further explored. In the future work, we will continue to explore the cardiovascular complications caused by blood glucose fluctuation and its internal mechanism.

## Conclusion

Collectively, our study suggests that both *in vivo* and *in vitro*, glucose fluctuation triggers activation of ER stress and promotes cardiomyocyte apoptosis more severely than sustained hyperglycemia. Meanwhile, 4-PBA can attenuate glucose fluctuation-induced cardiomyocyte apoptosis by inhibiting the ER stress signaling pathway. Thus, ER stress can potentially be a useful therapeutic target for diabetic cardiomyopathy.

## Supplementary Material

Supplemental Material

## Data Availability

The data that support the findings of this study are available from the corresponding author upon reasonable request.
