# Supplementary material for: Glucose fluctuation promotes cardiomyocyte apoptosis by triggering endoplasmic reticulum (ER) stress signaling pathway in vivo and in vitro
Source: Bioengineered. 2022 Jun 15;13(5):13739–51. doi: 10.1080/21655979.2022.2080413 (PMC9275931; doi:10.1080/21655979.2022.2080413)
Supplement: Supplemental Material [file KBIE_A_2080413_SM3308.pdf]

实验动物福利伦理审查同意书

Affidavit of Laboratory Animal Ethics and Welfare review

申请编号<br>Application No.		批准编号<br>Approval No.	

本《动物实验方案》经过实验动物伦理委员会审核，符合动物保护、动物福利和伦理原则，符合国家实验动物福利伦理的相关规定。方案的相关信息如下：

The animal use protocol listed below has been reviewed and approved by the Institutional Animal Care and Use Committee（IACUC）.

实验名称<br>Protocol Title	血糖波动通过蛋白激酶C/核因子κB/肌环指蛋白1信号通路对冠状动脉平滑肌细胞BK通道的损伤作用及其调控机制<br>The injurious effects and regulatory mechanisms of glucose fluctuation on BK channels of coronary arterial smooth muscle cells via PKC/NF-κB/MuRF1 signaling pathway				
申请人姓名<br>Applicant	王如兴<br>Ru-Xing Wang	职称/学位<br>Title/Degree	教授<br>Professor	邮箱<br>Email	ruxingw@aliyun.com
实验负责人<br>Principle Investigator	王如兴<br>Ru-Xing Wang	职称/学位<br>Title/Degree	教授<br>Professor	邮箱<br>Email	ruxingw@aliyun.com
部门<br>Department	南京医科大学附属无锡人民医院<br>The Affiliated Wuxi People’s Hospital of Nanjing Medical University			申请日期<br>Application Date	2017-12-01
拟实验时间<br>Period of Protocol	2018-1-1至<br>2021-12-31	动物实验设施许可证编号<br>No.of Animal Use License		SYXK（苏）2015-0004	
审核意见Results of Inspection	☐ 符合动物福利伦理要求，可以进行实验。 Agree<br>☐ 调整方案后，可以进行实验。 Agree after modify				
兽医师<br>Chief Veterinary Officer				日期<br>Date	

南京医科大学实验动物福利伦理委员会
Institutional Animal Care and Use Committee of NJMU

主席(Chairman)：

日期(Date)：

实验动物福利伦理审查同意书
Affidavit of Laboratory Animal Ethics and Welfare review

申请编号<br>Application No.	/	批准编号<br>Approval No.	IACUC-1712028

本《动物实验方案》经过实验动物伦理委员会审核，符合动物保护、动物福利和伦理原则，符合国家实验动物福利伦理的相关规定。方案的相关信息如下：

The animal use protocol listed below has been reviewed and approved by the Institutional Animal Care and Use Committee（IACUC）.

实验名称<br>Protocol Title	血糖波动通过蛋白激酶C/核因子κB/肌环指蛋白1信号通路对冠状动脉平滑肌细胞BK通道的损伤作用及其调控机制<br>The injurious effects and regulatory mechanisms of glucose fluctuation on BK channels of coronary arterial smooth muscle cells via PKC/NF-κB/MuRF1 signaling pathway				
申请人姓名<br>Applicant	王如兴<br>Ru-Xing Wang	职称/学位<br>Title/Degree	教授<br>Professor	邮箱<br>Email	ruxingw@aliyun.com
实验负责人<br>Principle Investigator	王如兴<br>Ru-Xing Wang	职称/学位<br>Title/Degree	教授<br>Professor	邮箱<br>Email	ruxingw@aliyun.com
部门<br>Department	南京医科大学附属无锡人民医院<br>The Affiliated Wuxi People's Hospital of Nanjing Medical University			申请日期<br>Application Date	2017-12-01
拟实验时间<br>Period of Protocol	2018-1-1至<br>2021-12-31	动物实验设施许可证编号<br>No.of Animal Use License		SYXK（苏）2015-0004	
审核意见Results of Inspection	☐ 符合动物福利伦理要求，可以进行实验。 Agree<br>☐ 调整方案后，可以进行实验。 Agree after modify				
兽医师<br>Chief Veterinary Officer				日期<br>Date	

南京医科大学实验动物福利伦理委员会
Institutional Animal Care and Use Committee of NJMU

主席(Chairman)：

日期(Date)：

南京医科大学实验动物福利伦理审查表

申请编号 Appl. No.	IACUC-1712028
申请日期 Appl. Date	2017-12-01

一、申请者基本情况

实验名称 Protocol Title	血糖波动通过蛋白激酶C/核因子κB/肌环指蛋白1信号通路对冠状动脉平滑肌细胞BK通道的损伤作用及其调控机制				
	The injurious effects and regulatory mechanisms of glucose fluctuation on BK channels of coronary arterial smooth muscle cells via PKC/NF-κB/MuRF1 signaling pathway				
项目来源及编号 Sponsor and No. of program	国家自然科学基金<br>81770331		拟实验时间 Experimental Period	2018-1-1至2021-12-31	
申请人姓名 Applicant	王如兴<br>Ru-Xing Wang	职称/学位 Title/Degree	教授<br>Professor	电话 Tel	15261530030
院系（部门） Department	南京医科大学附属无锡人民医院<br>The Affiliated Wuxi People’s Hospital of Nanjing Medical University			邮箱 Email	ruxingw@aliyun.com
实验负责人 Principal Investigator	王如兴<br>Ru-Xing Wang	职称/学位 Title/Degree	教授<br>Professor	电话 Tel	15261530030
院系（部门） Department	南京医科大学附属无锡人民医院<br>The Affiliated Wuxi People’s Hospital of Nanjing Medical University			邮箱 Email	ruxingw@aliyun.com

二、实验人员（没有资格证号不予受理）

实验执行人	职称/学位	动物实验资格证编号	电话	邮箱
张桢烨	硕士	2170692	18795888160	506890767@qq.com

三、项目信息

3.1 本研究的目的及科学贡献（以非专业语言简述，≤1000字）

目前越来越多研究证实血糖波动比持续性高血糖更能促使糖尿病血管并发症的发生，但其机制不明。申请者在主持的前一个已结题的国家自然科学基金课题研究中发现糖尿病时冠状动脉平滑肌细胞BK通道β1-亚单位表达减少，在本项目前期工作研究中，申请者发现血糖波动可使冠状动脉平滑肌细胞PKC活性增强，NF-κB磷酸化水平升高，MuRF1蛋白表达增加，BK通道β1-亚单位表达减少。据此提出如下科学假说：血糖波动通过PKC/NF-κB/MuRF1信号通路降低冠状动脉平滑肌细胞BK通道β1-亚单位表达，从而使BK通道功能受损。为验证这一假说，申请者拟通过建立血糖波动模型，采用膜片钳、分子生物学和血管张力测定等实验技术，从离子通道、细胞、组织及动物整体水平多层次探讨血糖波动对BK通道的损伤作用及其调控机制，为揭示血糖波动对BK通道的损伤作用机制奠定基础，研究结果可能为糖尿病及其并发症的进一步研究提供理论依据。

3.2 主要参考文献（≤5篇）

Saito S, Teshima Y, Fukui A, Kondo H, Nishio S, Nakagawa M, Saikawa T, Takahashi N. Glucose fluctuations increase the incidence of atrial fibrillation in diabetic rats, Cardiovasc Res, 2014 Oct 1;104(1):5-14. doi: 10.1093/cvr/cvu176.

3.3 本项目是否与已通过审查的项目相关联（重复或类同）？如果有，具体的项目名称与受理号并附加说明：

四、动物信息

动物来源	□ 繁育中心<br>□ 五台动物房<br>□ 基地公用平台动物房<br>☑ 国内其他饲养繁殖单位<br>□ 国外引进	质量合格证	☑ 有<br>□ 无
品种、品系	☑ 大鼠<br>□ 小鼠<br>□ 裸鼠<br>□ 其他（具体说明）	等级	□ 普通<br>□ 清洁<br>☑ SPF<br>□ 无菌
数量	♀____ ♂ 200	周龄	6-8周
饲养设施	□ 五台动物房<br>□ 江宁动物房<br>☑ 其他（请填写校外实验动物设施使用证明）SYXK（苏）2015-0004	设施类型	☑ 屏障设施<br>□ 普通设施<br>□ 隔离设施
动物使用的理由	□ 一些生物学过程和机理不能在体外研究<br>☑ 已进行体外实验，现必须进行体内实验<br>□ 体外实验需要动物组织<br>□ 其它，请具体说明：		

动物品种选择的理由	☑ 该品种的生理学、解剖学、身体大小等特点最适于本研究<br>☑ 该品种是本实验公认的理想动物模型<br>□ 利用该品种已获得大量的相关数据，本研究进一步扩展该品种相关数据<br>□ 从其它品种动物扩展相关数据到该品种<br>□ 其它，请具体说明：
动物实验计划与分组	采用链脲霉素腹腔内注射建立糖尿病大鼠动物实验模型。采用胰岛素注射法建立糖尿病大鼠血糖波动动物实验模型，以血糖浓度峰值>22.2mmol/L，谷值<5.5mmol/L作为判断血糖波动的标准。血糖波动动物实验分正常血糖组、高血糖组和血糖波动组三组。

五、实验信息

5.1 动物实验主要观察指标

大鼠冠状动脉平滑肌细胞BK 通道全细胞和单通道电流记录；
大鼠冠状动脉血管张力；

5.2 给药方案（包括注射细胞）

药名	给药剂量	给药频率	给药途径	给药部位
链脲霉素	60 mg/kg	一次	腹腔注射	腹腔

5.3 标本采集方案

采集的组织或体液	采集方法	数量或体积	采集频率	持续时间或最大采集数量
静脉血	下腔静脉采血	5ml	1次	5ml
冠状动脉平滑肌细胞	酶消化法	500μl	1次	500μl

5.4 手术操作描述　（≤1000字）

术者技术资质	无	手术地点	无
手术后护理	无	同一动物上进行多个操作	□ 否　□ 是
疼痛评价	□ 无疼痛或痛苦<br>□ 轻微疼痛或痛苦<br>□ 明显疼痛或痛苦<br>□ 重度疼痛或痛苦		

手术描述：

无

5.5　麻醉与镇痛

药物名称及浓度	给药剂量和频率	给药途径	维持时间
异氟醚	2%	气体吸入	10分钟

5.6　实验动物生命终结标准

□ 1、体重减轻：体重减轻达 20-25%，或是动物出现恶病质或消耗性症候时。

□ 2、实体瘤的大小超过动物体重的 10%。

□ 3、丧失食欲：小型啮齿类动物完全丧失食欲达 24 小时或食欲不佳（低于正常量之50%） 达 3 天时。大动物完全丧失食欲达 5 天或食欲不佳（低于正常量之 50%） 达 7 天时。

☑ 4、虚弱（无法进食或饮水）：动物在没有麻醉或镇静的状态下，无法进食或饮水， 长达 24 小时无法站立或极度勉强才可站立时。

□ 5、垂死/濒死：动物在没有麻醉或镇静的状态下，表现精神抑郁伴随体温过低（低 于 37℃）时。

□ 6、感染，在抗生素治疗无效并伴随动物全身性不适症状。

☑ 7、器官：出现器官严重丧失功能的临床症状且治疗无效，或经动物中心兽医师判 断预后不佳。

☑ 8、呼吸系统：呼吸困难、发绀大失血。

□ 9、心血管系统：大失血、已给予一次输液治疗后仍贫血（低于 20%）。

□ 10、消化系统：严重呕吐或下痢，消化道阻塞，套迭，腹膜炎，内脏摘除手术。

□ 11、神经系统：中枢神经抑制、震颤、瘫痪（其中任一肢或以上）、对止痛剂治疗 无效之疼痛。

□ 12、肌肉骨骼系统：肌肉受损或骨折使肢体丧失功能（实验预期发生并通过 IACUC 审核除外）。

□ 13、皮肤：无法治愈之伤口、重复性自残或二级以上之保温垫烫伤。

5.7 动物安乐死方法

	小于125g 啮齿动物	125g～1kg 啮齿动物/兔	1kg～5kg 啮齿动物/兔	犬	猫	非人灵长类	牛、马、猪	两栖类/鱼类
□二氧化碳	√	√	√	X	X	X	X	√
□巴比妥钠静脉注射（100 mg/ kg）	√	√	√	√	√	√	√	√
☑巴比妥钠腹腔注射（100 mg/ kg）	√	√	√	X	√	X	√	√
□ 麻醉后放血致死	√	√	√	√	√	√	√	√
□ 麻醉后静脉注射 Kcl（1-2 mg/ kg）	√	√	√	√	√	√	√	√
□ 麻醉后断头	√	√	△	X	X	X	X	√
□ 麻醉后颈椎脱位	√	√	X	X	X	X	X	X
□ 动物清醒中直接断头	△	△	△	X	X	X	X	X
□ 动物清醒中直接颈椎脱位	△	X	X	X	X	X	X	X
□ 乙醚	△	X	X	X	X	X	X	X

表格说明：√ 建议使用之方法；× 不建议使用之方法；△ 说明理由并经动物实验管理委员会审核通过后可使用之方法。

5.8用CO2或其他吸入性气体对动物安乐死时，需准备另一种物理方法，在动物无意识的状态下，对其进一步安乐死

□ 1、断头
□ 2、头颈部脱臼
□ 3、放血法，如割开大血管、心脏灌注、摘取主要器官
□ 4、切开胸部，使动物气胸和停止呼吸
□ 5、其它 ，请具体说明:

5.9用过剂量的药物（如戊巴比妥）对动物实施安乐死时，在给药10分钟后必须再次检测动物的生命体征。采用以下标准判断动物已死亡

☑ 1、动物已经不动了，没有呼吸了
☑ 2、动物已没有呼吸、心跳、角膜反射、肌张力和粘膜颜色
□ 3、其它 ，请具体说明：

5.10 实验后动物处理

☑ 1、安乐死
□ 2、返回供应单位
□ 3、转给其它课题组
□ 4、饲养直至其自然死亡
□ 5、其它 ，请具体说明:

5.11 动物尸体、组织或体液的处理

□ 1、制作标本
☑ 2、集中无公害化处理
□ 3、其它 ，请具体说明:

5.12 动物实验中使用的试剂

□ 1、放射性同位素
□ 2、生物物品
☑ 3、化学品、药品
□ 4、重组
□ 5、其它

5.11 如对伦理审查有特殊要求，请说明(例如需要某一委员回避等)

承诺书

我同意遵守中华人民共和国国家科学技术委员会制定的《实验动物管理条例》、中华人民共和国科学技术部发布的《关于善待实验动物的指导性意见》、江苏省人民政府发布的《江苏省实验动物管理办法》。

我承诺该申请表的内容真实准确，提及的实验人员已经参加实验动物相关培训，掌握实验中涉及的动物实验方法，深知使用这些活体动物及物组织所存在的风险。

我将自觉遵守实验动物福利伦理原则，同意接受委员会或实验室管理者的监督与检查。

实验负责人：[illegible]

实验执行人（包括参与实验的所有人员）：

[illegible] 钱[illegible] 张[illegible] [illegible] 刘晓[illegible] 2017年12月1日

审查依据	1、该实验是否必须用实验动物进行实验，即能否用计算机模拟、细胞培养等非生命方法替代动物或用低等动物替代高等动物进行实验<br>2、表中所填实验相关人员资格和实验相关单位是否合适。<br>3、表中所填实验所用动物能否通过改良设计方案或用高质量的动物来减少所用 动物的数量。<br>4、能否通过改进实验方法、调整实验观测指标、改良处死动物的方法，来优化实验方案、善待动物。<br>5、实验设计、实验技术方法及用于本实验的动物数量是否合理可行。

实验动物福利伦理委员会审查意见：<br><br>委员签字（签章）： 年 月 日
备注

说明：

1、申请表须在实验前 2 个月提交。要求写明课题的意义、必要性，表中有关实验动物的用 途、饲养管理或动物实验操作和观察步骤、实验终结标准、减少动物痛苦伤害措施的程序和方法等涉及动物福利伦理问题的需要详细描述，可以增加附页。

2、登陆http://iacuc.njmu.edu.cn:8080进行用户注册,注册激活后可在动物伦理申请栏,进行申请书填写、初审意见查看、申请书修改、打印及对历史申请进行管理。

3、申请书首先经过伦理委员会初审,请根据初审意见修改并提交，然后再经初审、复审。 通过后将本申请表下载并单面打印，课题负责人、执行人及合作单位负责人均需在声明人签字栏签字。然后连同必要的审查资料递交到动物伦理委员会秘书处。

4、**需在外单位完成的课题，请同时填写院外实验动物设施使用证明。 凡项目执行人无实验动物上岗培训合格证明材料（需在有效期内）的课题不能进行，亦不做伦理审查。**

5、申请人如对结果有异议，请与动物伦理委员会联系；如需要复议可以在提交新的材料后申请动物伦理委员会复议。联系电话：张老师，025-86867156。
